# Spatial Information of Somatosensory Stimuli in the Brain: Multivariate Pattern Analysis of Functional Magnetic Resonance Imaging Data

**DOI:** 10.1155/2020/8307580

**Published:** 2020-06-29

**Authors:** In-Seon Lee, Won-mo Jung, Hi-Joon Park, Younbyoung Chae

**Affiliations:** Acupuncture & Meridian Science Research Center, College of Korean Medicine, Kyung Hee University, Seoul, Republic of Korea

## Abstract

**Background:**

Multivoxel pattern analysis has provided new evidence on somatotopic representation in the human brain. However, the effects of stimulus modality (e.g., penetrating needle versus non-penetrating touch) and level of classification (e.g., multiclass versus binary classification) on patterns of brain activity encoding spatial information of body parts have not yet been studied. We hypothesized that performance of brain-based prediction models may vary across the types of stimuli, and neural patterns of voxels in the SI and parietal cortex would significantly contribute to the prediction of stimulated locations.

**Objective:**

We aimed to (1) test whether brain responses to tactile stimuli could distinguish among stimulated locations on the body surface, (2) investigate whether the stimulus modality and number of classes affect classification performance, and (3) localize brain regions encoding the spatial information of somatosensory stimuli.

**Methods:**

Fifteen healthy participants completed two functional magnetic resonance imaging (MRI) scans and were stimulated via the insertion of acupuncture needles or by non-invasive touch stimuli (5.46-sized von Frey filament). Participants received the stimuli at four different locations on the upper and lower limbs (two sites each) for 5 min while blood-oxygen-level-dependent activity (BOLD) was measured using 3-Tesla MRI. We performed multivariate pattern analysis (MVPA) using parameter estimate images of each trial for each participant and the support vector classifier (SVC) function, and the prediction accuracy and other MVPA outcomes were evaluated using stratified five-fold cross validation. We estimated the significance of the classification accuracy using a permutation test with randomly labeled training data (*n* = 10,000). Searchlight analysis was conducted to identify brain regions associated with significantly higher accuracy compared to predictions based on chance as obtained from a random classifier.

**Results:**

For the four-class classification (classifying four stimulated points on the body), SVC analysis of whole-brain beta values in response to acupuncture stimulation was able to discriminate among stimulated locations (mean accuracy, 0.31; *q* < 0.01). The searchlight analysis found that values related to the right primary somatosensory cortex (SI) and intraparietal sulcus were significantly more accurate than those due to chance (*p* < 0.01). On the other hand, the same classifier did not predict stimulated locations accurately for touch stimulation (mean accuracy, 0.25; *q* = 0.66). For binary classification (discriminating between two stimulated body parts, i.e., the arm or leg), the SVC algorithm successfully predicted the stimulated body parts for both acupuncture (mean accuracy, 0.63; *q* < 0.001) and touch stimulation (mean accuracy, 0.60; *q* < 0.01). Searchlight analysis revealed that predictions based on the right SI, primary motor cortex (MI), paracentral gyrus, and superior frontal gyrus were significantly more accurate compared to predictions based on chance (*p* < 0.05).

**Conclusion:**

Our findings suggest that the SI, as well as the MI, intraparietal sulcus, paracentral gyrus, and superior frontal gyrus, is responsible for the somatotopic representation of body parts stimulated by tactile stimuli. The MVPA approach for identifying neural patterns encoding spatial information of somatosensory stimuli may be affected by the stimulus type (penetrating needle versus non-invasive touch) and the number of classes (classification of four small points on the body versus two large body parts). Future studies with larger samples will identify stimulus-specific neural patterns representing stimulated locations, independent of subjective tactile perception and emotional responses. Identification of distinct neural patterns of body surfaces will help in improving neural biomarkers for pain and other sensory percepts in the future.

## 1. Introduction

The somatosensory system is distributed throughout the human body and responds to noxious and innocuous sensations, whereas the brain processes multiple sensory factors influencing the perception of somatosensory stimuli such as intensity [1–3], modality [4, 5], and location [6–8]. Extensive research has identified brain regions involved in the processing of stimulus intensity, subjective intensity, emotional responses, and the cognitive evaluation of pain and non-painful somatosensory stimuli, namely, the primary and secondary somatosensory cortexes (SI and SII, respectively), anterior cingulate cortex, thalamus, and insula [9–11].

Studies on the neural processing of spatial information of somatosensory stimuli have mainly focused on the somatotopic representation of body surfaces [6, 12–15] since Penfield and Boldray reported their landmark study of a motor and sensory homunculus in the human brain [16]. In addition, effects of the location or posture of the stimulated body part on the perception of stimulus location (spatial remapping from skin into external space [17, 18]) and brain activations related to the spatial discrimination task [7, 19, 20] have been studied. The major candidates subserving perception and discrimination of spatial information of somatosensory stimuli in the brain are the SI [6, 12, 21] and posterior parietal cortex [18, 22, 23]. The SI reflects a distinct somatotopic representation of body parts; for example, the lower limb is represented in the medial-superior part of the SI, whereas the upper limb is represented in the lateral part. The posterior parietal cortex updates the representation of the body in external space by integrating proprioceptive information and sensory inputs and is thus critical in spatial remapping.

Recently, machine learning and multivariate pattern analysis (MVPA) have been extensively used in functional magnetic resonance imaging (fMRI) data analysis as an alternative to conventional univariate analyses. In a previous study, MVPA was able to distinguish among the locations of vibrotactile stimulation delivered to widely separated parts of the body (e.g., bilateral hands and feet; predicted correctly for 85% of trials) or to closely located parts of the body (e.g., three digits of the same hand) [8]. Moreover, the SI and SII exhibited better decoding performance than other regions of interest (ROIs), with an average accuracy of 60% for widely separated body parts (chance accuracy, 25%). Mean classification accuracies for three-way decoding of three digits of the same hand by the SI and SII were 61% and 50% (chance accuracy, 33%), respectively, which were better than those exhibited by other ROIs. For vibrotactile stimuli delivered to a single digit, high-resolution 7-Tesla fMRI revealed location-specific activations in the SI, and using neural signals of the index finger-associated region in the SI, MVPA successfully differentiated the location of four stimulated sites within a single digit (mean accuracy, 65%) [24]. In our previous study, we tested the ability of MVPA to discriminate two adjacent sites on the arm receiving pricking pain sensations induced by an acupuncture needle and found that the SI, primary motor cortex (MI), insula, and inferior parietal cortex participated in predicting the location of the pain [20].

The results of previous studies suggest that the SI and SII, as well as other somatosensory processing regions, contain informative multivariate patterns predicting the location of exteroceptive stimuli applied to the human body. However, it remains unclear whether the distinguishable patterns encoding spatial information in the brain vary depending on the stimulus modality (i.e., penetrating needle versus nonpenetrating touch) and number of classification classes (e.g., multiclass versus binary classification). Based on the previous findings, we hypothesized that (1) the accuracies of brain-based prediction models may vary across the types of stimuli (however, we had no prior hypothesis about which model would predict stimulated locations better than the other one due to insufficient evidence) and (2) clusters of significant voxels identified by searchlight analysis are scattered predominantly in the SI and parietal cortex.

To test our hypotheses, we (1) investigated whether whole-brain responses to two tactile stimuli, one painful and one non-painful, could predict stimulated locations on the body, (2) investigated whether the stimulus modality and number of classes affect classification performance, and (3) identified brain regions encoding the somatotopic information of somatosensory stimuli.

## 2. Methods

### 2.1. Participants

Fifteen healthy participants (mean age, 22.8 years; six females) underwent two fMRI scans. All participants were not naïve to acupuncture, and they were refrained from alcohol or caffeine for 12 h before the experiments. Participants were not enrolled if they had any skin problems on the stimulated site or if they were not eligible for MRI (e.g., suffer from claustrophobia). After the experimental procedure was explained, all participants provided written consent. This study was carried out in accordance with the guidelines for clinical research established by the Kyung Hee University Ethics Committee (Seoul, Republic of Korea), and it was also approved by the Kyung Hee University Ethics Committee (KHSIRB-15-056).

### 2.2. Study Design and Experimental Procedures

**Figure 1:**
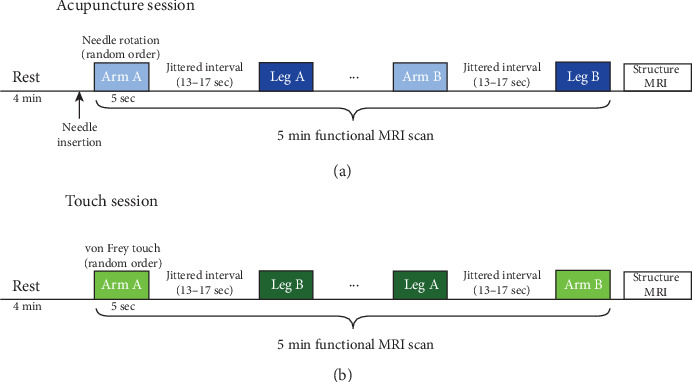
Illustration of experimental procedure. Two fMRI sessions were performed on separate days, with at least 5 days off in between, while participants received either (a) acupuncture or (b) von Frey touch stimulation inside of the scanner. Two sites each on the arm (Arm A and Arm B) and the leg (Leg A and Leg B) were stimulated in a random order while the blood-oxygen-level-dependent signal was measured. In the acupuncture session, acupuncture needles were inserted into the four acupoints each after a 4-minute resting scan, and one of the needles was rotated at a rate of 1 Hz according to the randomized order. In the touch session, a 5.46-sized von Frey filament was applied to one of the stimulation sites at a rate of 1 Hz according to the randomized order. Each stimulation was delivered for 5 seconds with jittered intervals (range 13-17 seconds) between trials. The structure image was obtained on day 2 at the end of the second session.

All participants completed two event-related fMRI sessions on two separate days, and the order of sessions was counterbalanced across participants. Each session was separated by at least 5 days and conducted at a similar time of day. Each session included a 4 min baseline resting state scan (not analyzed in this study) followed by a 5 min stimulation scan. During the stimulation scan, participants experienced 20 trials of an acupuncture or tactile stimulus at four locations, and the order of stimulated locations was randomized in each participant (5 s for each stimulus with jittered intervals of 14.92 ± 1.66 s between trials). After the stimulus scan, another fMRI scan using the histamine-induced itch paradigm was acquired, and the results were reported elsewhere (see Min et al. [25]). Anatomical images were acquired at the end of the session (Figure 1).

### 2.3. Somatosensory Stimulation

In each session, participants received acupuncture or were stimulated using a von Frey filament at four sites on their body. The acupuncture needle and von Frey filament were used to evoke pain and touch sensations, respectively. All stimulations were performed by a licensed Korean medicine doctor. For the acupuncture session, two sites each on the left arm (Arms A and B: acupoints PC6 and HT7) and leg (Legs A and B: acupoints SP10 and ST36) were stimulated. Non-magnetic titanium acupuncture needles (0.20 mm in diameter and 40 mm in length; DongBang Acupuncture Inc., Boryeong, Republic of Korea) were inserted into the four acupoints at a safe depth for each acupoint (6 mm for HT7, 15 mm for SP10 and PC6, and 20 mm for ST36) before beginning the stimulation scan. During the scan, one of the inserted needles was rotated bidirectionally at a rate of 1 Hz guided by a metronome sound transmitted via headphones according to the randomized order.

Tactile stimulation was delivered by gentle tapping of an MRI-compatible von Frey filament (size 5.46, target force 26 g; Touch Test Sensory Evaluator Instructions, North Coast Medical, Inc., Morgan Hill, CA, USA) at a rate of 1 Hz guided by the metronome sound. The locations were labeled as same as the acupuncture session (Arm A, Arm B, Leg A, and Leg B), although they were different from the acupoints used for acupuncture stimulation. Tactile stimulation sites were located on the same meridian system but are not acknowledged as acupoints. Detailed information was described in our previous study [25].

### 2.4. fMRI Data Acquisition and Analysis

Data were acquired on a 3-Tesla MRI scanner (Siemens, Erlangen, Germany) with a three-axis gradient head coil. Functional images were acquired with a T2∗-weighted gradient echoplanar imaging sequence (37 slices; TR = 2,000 ms; TE = 30 ms; flip angle, 90°; field of view, 240 × 240 mm^2^; slice thickness, 4.0 mm; voxel size = 3 mm × 3 mm × 3 mm^3^). Anatomical images were acquired using a T1-weighted rapid gradient echo sequence with TR = 2,000 ms, TE = 2.37 ms, a flip angle of 9°, a field of view of 240 × 240 mm^2^, and a slice thickness of 1.0 mm.

Preprocessing of the fMRI data was performed using Analysis of Functional NeuroImages (AFNI version 19.2.24, https://afni.nimh.nih.gov) [26]. The echo-planar imaging data were despiked, corrected for slice time differences, realigned for motion correction, coregistered to the individual anatomical image, and scaled voxel-wise using the mean value of each voxel. Trial-specific estimates were obtained using a beta-series regression, least squares model, modelling each trial as a separate regressor constructed by convolving a gamma function with a box-car function at the onset of each trial [27]. We made a beta image for each scan separately (acupuncture and tactile) and used two different sets of labels for the stimulated locations. One trial-specific beta image was computed using the trial labels of all stimulated locations (four-class; Arm A, Arm B, Leg A, and Leg B), and another beta image was made using labels of two body parts (two-class; Arm A and Arm B were labeled as “upper limb,” and Leg A and Leg B were labeled as “lower limb”). The resulting parameter estimates were used as input features for MVPA.

### 2.5. MVPA and Statistical Analysis

**Figure 2:**
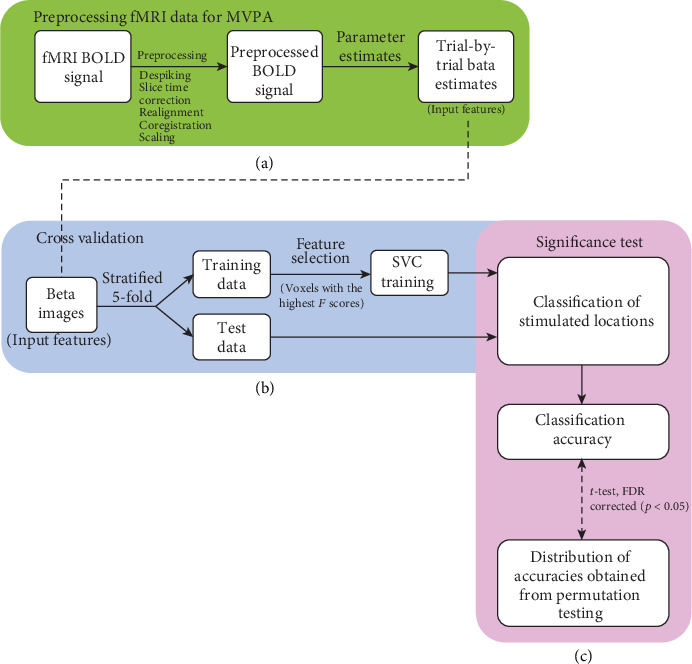
Flowchart of the MVPA method. (a) Raw fMRI data were preprocessed with AFNI (version 19.2.24) including despiking, slice time correction, realignment for motion correction, coregistration to the individual anatomical image, and scaling voxel-wise signal using the mean value of each voxel. Trial-specific estimates (beta images) were obtained using the least squares regression, which estimates each trial separately by convolving a gamma function with a box-car function. The outputs (parameter estimates) were used as input features for the MVPA. (b) Training and test data sets were generated, and a stratified 5-fold cross validation was performed. The SVC was trained with the top 10 percentile of features with the highest *F* scores. (c) We tested the SVC's prediction performance using the prediction accuracy, the area under the receiver operating characteristic curve, recall, precision, and F1 score, and the accuracy was compared to a permutation test with randomly labeled training data (*n* = 10,000). AFNI: Analysis of Functional NeuroImages; BOLD: blood-oxygen-level-dependent; FDR: false discovery rate; fMRI: functional magnetic resonance imaging; MVPA: multivariate pattern analysis; SVC: support vector classifier.

The multivariate analysis was performed using the Python package scikit-learn (Figure 2) [28]. We applied the support vector classifier (SVC) function with a linear kernel to predict the stimulated locations, and the one-vs.-one scheme was employed for the four-class classification. *F* score-based feature selection was performed on the training data, and 10 percentile of the voxels with the highest *F* scores were included as input features. The classification accuracy for each voxel in the brain (whole-brain classification accuracy) was estimated as its generalization ability with a stratified five-fold cross validation procedure. In addition, we estimated the performances of the classifiers as the area under the receiver operating characteristic curve (ROC-AUC), precision, recall, and F1 score, and summarized the correct and incorrect classifications of the target locations using the confusion matrix.

The significance of the classification accuracies of all voxels was tested using a non-parametric random permutation test (*n* = 10,000) and paired *t*-tests, and results were corrected for multiple comparisons using the false discovery rate (FDR) approach (the significance threshold was set at *q* < 0.05). Neural patterns encoding stimulated locations were localized using spherical multivariate searchlight analysis (radius = 5 mm) [29]. Searchlight results were identified using an uncorrected voxel-wise threshold of *p* < 0.001 and corrected for multiple comparisons using the Monte Carlo simulation at the corrected significance threshold of *p* < 0.05 [30].

## 3. Results

### 3.1. Whole-Brain SVC Classification Performance

**Figure 3:**
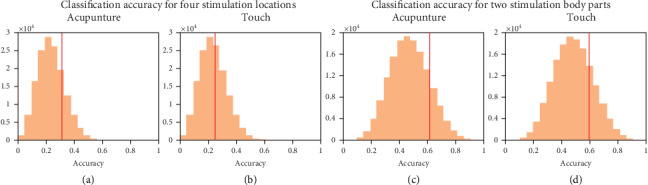
Classification performance using the support vector classifier (SVC) tool to discriminate among stimulated locations on the body surface. The mean accuracy of the SVC (red vertical lines) on whole-brain multivariate activity patterns and distribution of accuracies from permutation tests (orange histogram bars) for acupuncture (a, c) and tactile stimulation trials (b, d). Classification accuracy was estimated using a stratified five-fold cross validation procedure. In the permutation test for the four-class classification, labels of four stimulation locations (Arm A, Arm B, Leg A, and Leg B) were randomized, and stratified five-fold cross validation was applied 10,000 times for each participant (left box (a, b)). For binary classification, the two acupoints on the upper limb (Arm A and Arm B) and those on the lower limb (Leg A and Leg B) were collectively labeled as “upper limb” and “lower limb,” respectively, and the cross validation and permutation test were performed in the same way as for the two-class classification (right box (c, d)). SVC classification accuracy was significantly higher than the null distribution of the permutation test results for predicting the four stimulated locations in the acupuncture stimulation trials (a), the two large body parts stimulated by acupuncture (c), and the two large body parts stimulated by a von Frey filament (d). However, using the same approach, significantly better classification performance compared to the null distribution was not observed in terms of predicting the four stimulated locations in the von Frey filament-stimulation trials (b).

**Figure 4:**
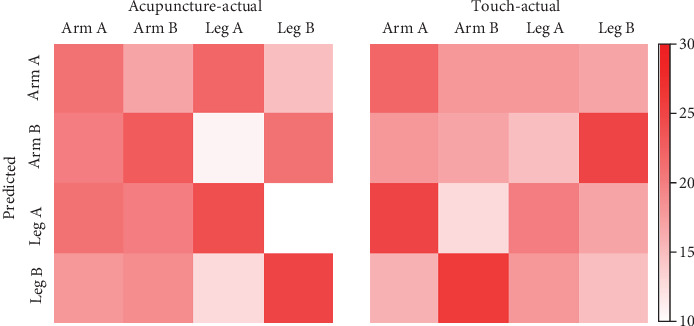
Confusion matrices for the four-way classification. The rows indicate the actual locations, and the columns indicate the predicted locations. The color bar presents the total number of predictions.

Classification accuracies of all voxels were compared to the distribution of accuracies from the permutation test to assess the classifier's performance in discriminating locations of the two types of somatosensory stimuli—acupuncture and touch stimuli. For the four-class classification (predicting one of four stimulated locations at a chance level of 0.25), classification with the MVPA analysis using whole-brain beta images and SVC was significantly more accurate in terms of predicting acupuncture-stimulated locations than the permuted accuracies obtained using randomized labels (mean accuracy, 0.31, FDR adjusted *q* < 0.01, Figure 3(a)). On the other hand, the same classifier was not significantly more accurate in terms of predicting tactile-stimulation locations compared to permuted accuracies (mean accuracy, 0.25, FDR adjusted *q* = 0.66, Figure 3(b)). The confusion matrix presents the frequencies of all classifications (Figure 4).

The same procedure was conducted to predict one of two stimulated body parts, the upper or lower limb (chance level, 0.5). The classifiers' prediction performance was significantly better than that of the permutation tests for both the acupuncture trials (mean accuracy, 0.63, FDR adjusted *q* < 0.001, Figure 3(c); ROC-AUC 0.63; precision estimate 0.63; recall estimate 0.63; mean F1 score 0.63) and the tactile stimulation trials (mean accuracy, 0.60, FDR adjusted *q* < 0.01, Figure 3(d); ROC-AUC 0.59; precision estimate 0.61; recall estimate 0.61; mean F1 score 0.60).

### 3.2. Decoding the Spatial Information of Somatosensory Stimuli on the Body

**Figure 5:**
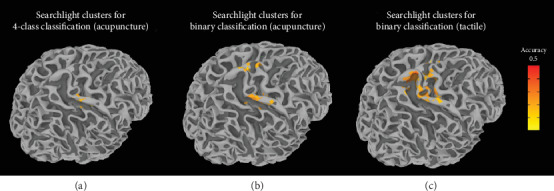
Searchlight analysis results. (a) Whole-brain searchlight analysis showed that the right SI and intraparietal sulcus allowed for statistically significant discrimination among four stimulated sites (Arm A, Arm B, Leg A, and Leg B) in the acupuncture trials. (b) Analyses of neural patterns of the right SI, MI, paracentral cortex, and superior frontal gyrus resulted in significantly accurate predictions of which of two body parts (leg or arm) was stimulated by acupuncture. (c) Analysis of neural patterns of the right SI, MI, superior parietal gyrus, paracentral cortex, and superior frontal gyrus resulted in significantly accurate predictions of which of two body parts was stimulated by the von Frey filament. All clusters were identified using an uncorrected voxel-wise threshold of *p* < 0.001 and corrected for FWE at a significance threshold of alpha < 0.05. The cluster extent threshold was determined with 10,000 iterations of a Monte Carlo simulation. FWE: family-wise error; MI: primary motor cortex; SI: primary somatosensory cortex.

To define brain regions including informative voxels of somatotopic representations of body surfaces stimulated by a penetrating needle or a non-penetrating touch stimulus, a 5 mm spherical searchlight analysis was conducted. The searchlight analysis showed that SVC performed better than a random classifier in the right SI when it is used to discriminate four locations stimulated via acupuncture (corrected family-wise error [FWE], *p* < 0.01; Figure 5(a)). No voxels were associated with significantly higher accuracy when the SVC was used compared to a random classifier for predicting the four locations of tactile stimulation. For binary classification (distinguishing between the stimulated locations on the upper and lower limbs), using SVC to analyze images of the right SI, paracentral gyrus, and superior frontal gyrus for both acupuncture (Figure 5(b)) and tactile stimulation (Figure 5(c)) was more accurate than using a random classifier (corrected FWE, *p* < 0.05). Details are given in Supplementary Table 1.

## 4. Discussion

### 4.1. Decoding the Spatial Location of Two Tactile Stimulations

The first aim of this study was to determine whether MVPA based on neural patterns for tactile stimuli could distinguish among stimulated locations on the body surface. We found that the MVPA was able to decode the body sites stimulated by two types of somatosensory stimuli—acupuncture and a von Frey filament—during the binary classification of two large body parts (leg and arm). However, during the four-way classification, in which one location was predicted out of four much smaller stimulated sites (two points each on the left leg and arm), MVPA successfully predicted the acupuncture-stimulated sites only, whereas the tactile-stimulation locations were not predicted more accurately than by chance. In addition, the confusion matrices for the four-way classification showed that the classification performance was found to be poor for touch stimulus, and also, there were discrepancies between prediction tendencies of each location. For example, the locations on the leg were more likely to be predicted accurately than those on the arm. The results suggest that the MVPA approach for neural patterns encoding somatotopic representation of somatosensations may be affected by the stimulus type (i.e., penetrating needle versus non-penetrating touch) and the number of classification tasks (i.e., four relatively small points on the body versus two relatively large body parts).

For the second aim of this study, we trained and tested classifiers on the data from two stimulation sessions, for both the binary and four-way classification tasks. In this study, the inputs to the classifiers were multivariate beta values across voxels from each participant. Thus, the performance of the classifier was determined by the neural responses to exteroceptive somatosensory stimuli. The locations of small body surfaces stimulated by the non-puncturing tactile stimulus were not predicted using neural patterns (i.e., classification predictions were not more accurate than predictions based on chance), whereas stimulated locations were successfully predicted using neural patterns responding to penetrating pain stimuli (i.e., more accurate than predictions based on chance). Although we could not compare the accuracies between the four-way classification (classifying four small points on the body) and the binary classification (classifying two large body parts), we found that both penetrating pain and non-penetrating touch stimuli produced distinguishable neural patterns that allow the determination of where the stimuli were delivered, either on the leg or the arm. This finding implies that MVPA classification performance in terms of locating somatosensation on the body surface might be altered by the stimulus modality and number of classes (the degree of classification difficulties).

### 4.2. Neural Representations of the Stimulated Body Parts

In agreement with previous studies of spatial information processing [8, 20, 24], MVPA provided detailed information of how stimulus locations are represented in the brain. Our searchlight results showed that neural activities in the SI, MI, posterior parietal cortex (superior parietal gyrus and intraparietal sulcus), paracentral gyrus, and superior frontal gyrus encode where they were stimulated. Interestingly, the clusters of informative voxels revealed by three successful classification tasks (four-way classification task for acupuncture stimulation and binary classification for acupuncture and von Frey filament stimulation) were overlapping but also divergent. The clusters carrying discriminative information for somatotopic representation of four different locations were relatively small (Figure 5(a)) and restricted to the SI and parietal area, whereas the clusters for distinguishing between two large body parts were largely distributed in the brain across the frontal and parietal cortices (Figure 5(b) and 5(c)). Binary classifiers for acupuncture (Figure 5(b)) and tactile stimulation (Figure 5(c)) were also represented by both overlapping and non-overlapping clusters in the brain, suggesting that different types of somatosensory stimuli may be processed in different local regions in terms of their somatotopic organization.

Previous studies found that stimulated fingers by vibrotactile stimulation and near-threshold painful stimuli can be decodable from brain patterns [31, 32]. Ritter et al. demonstrated that the MVPA approach can decode spatial information of pain on the arm or leg [33], and Beauchamp et al. found that MVPA was able to distinguish among the locations of vibrotactile stimulation delivered to widely separated parts of the body or to closely located parts of the body [8]. However, these studies mainly focused on single tactile modality, either painful or touch stimulus. To our best knowledge, this is the first study that tested and reported MVPA classifiers' performance for discriminating stimulated locations, both small areas and large regions of the body, when they were stimulated by two different tactile modalities.

### 4.3. Caveats, Limitations, and Implications

Our approach is the first attempt to reveal the modality-specific neural patterns of needle-induced pain different from non-invasive tactile stimulation, and the findings suggest a possible effect of stimulus modality on neural patterns encoding spatial information of the body. However, we need further studies in order to answer our questions about the effects of modality and level of classification, as we tested only two tactile modalities (penetrating needle versus non-penetrating touch) and two numbers of classes (2 versus 4) in this study.

The rationale for the options chosen for the MVPA should be described. We implemented SVC, which uses a linear kernel and one-vs.-one approach for multiclass classification. *k*-Nearest neighbor (KNN) and support vector machine (SVM) functions have been used for multiclass classifications, and we selected linear SVC, as previous studies have reported that SVM outperformed KNN [34]. We also used *F* score-based feature selection to reduce the number of input features, based on analysis of variance (ANOVA). A recent study by Xu et al. demonstrated that neural features selected based on their discrimination characteristic (e.g., ANOVA) exhibited better classification performance with higher prediction accuracy for distinguishing tasks or conditions than did features selected based on their reliability [35]. However, it should be noted that MVPA results are sensitive to combinations of parameters, and various combinations should be tested in the future.

The limitations of this study should be addressed. The sample size is not large enough to represent the population, and the small number of trials limits the generalizability of our results. In addition, due to the lack of subjective intensity ratings for the stimuli, we cannot conclude whether the regions represented the location of sensation (“where it hurts/feels touch”) or the location of stimulus (“where it is being stimulated”). An acupuncture needle stimulates the body in an invasive manner and evokes pricking pain. Conversely, the von Frey filament is non-invasive, but can produce painful or non-painful tactile sensations according to the target force and individual differences. In our previous study, acupuncture was associated with greater subjective intensity ratings compared with tactile stimulation when delivered to the same loci [36]. Thus, we assumed that an acupuncture needle would generate a painful sensation and a von Frey filament would provide a non-painful touch sensation to participants in this study. As subjective experience and emotional factors influence cortical responses to somatosensory stimulation, a function of the subjective experience of the stimulus should be investigated in future studies. In addition to the intensity, the distance between stimulated locations and cutaneous innervation might influence the prediction of stimulated locations. Lastly, understanding the cross-modal prediction of the classifier by testing the classifier, which was trained on the neural patterns evoked by one stimulus, on the neural patterns evoked by the other stimuli will be an important direction for future work. To achieve this goal, a large number of trials with random order of stimuli are necessary.

This work reveals the neural mechanisms underpinning the spatial tactile discrimination and demonstrates that neural patterns differently encode locations of different tactile stimuli. The central processing of spatial information of somatosensations (i.e., location of sensations on the body) has been less studied using the MVPA technique compared to other somatosensory factors such as functions of stimulus intensity or subjective intensity. However, identifying neural representations of the locations of stimulations or sensations is crucial for developing brain-based biomarkers for somatosensations such as pain. We are still relying on subjective reporting by patients to describe pain, although research on the biomarkers of pain has advanced greatly in past years. A few studies have demonstrated that neuroimaging-based patterns can be used to predict subjective pain experiences [37]. The patterns of somatotopic representation for exteroceptive touch stimulation (i.e., where the body is being touched) and subjective pain experience (i.e., where it hurts) will improve the current approach in the search for neural biomarkers of somatosensory percepts. For example, brain patterns encoding spatial information of somatosensations will allow us to locate where patients feel pain or other somatosensory percepts (e.g., itchiness, touch, and temperature). It will contribute to more precise and reliable clinical application of neuroimaging-based biomarkers. In addition, we could test hypotheses of whether neural patterns responsible for the spatial representation of somatic and visceral sensations are overlapping and whether chronic and acute pain experienced at the same location are distinguishable in future studies. Taken together, distinct neural representations of body sites will expand our understanding of somatotopic maps of our body in the brain, which will lead us to develop neuroimaging-based biomarkers for clinical improvements of pain in chronic pain patients.

## 5. Conclusions

The results from MVPA indicate that the SI, as well as the MI, intraparietal sulcus, paracentral gyrus, and superior frontal gyrus, encodes the spatial location of somatosensory stimuli. Moreover, the prediction performance of the MVPA classifier using neural patterns in response to somatosensory stimuli may be affected by the stimulus type (penetrating needle versus non-invasive touch) and the number of classification classes (classification of four small points on the body versus two large body parts). Future studies with larger samples are required to identify spatial patterns of brain activity reflecting stimulated locations independent of subjective intensity ratings, emotional states (e.g., anxiety and fear), and cognitive factors (e.g., attention, expectation, and appraisal). This approach will lead to the identification of brain multivariate patterns representing “where it hurts” and “where stimulation is felt” for various types of somatosensory stimuli and other somatic sensations, which will help in the development of neural biomarkers for pain and other sensory percepts.

## Supplementary Materials

Supplementary materials Table 1: Clusters identified in the searchlight analysis encoding stimulated locations for acupuncture and touch stimulation.
