# Supplementary material for: Spatial Information of Somatosensory Stimuli in the Brain: Multivariate Pattern Analysis of Functional Magnetic Resonance Imaging Data
Source: Neural Plast. 2020 Jun 29;2020:8307580. doi: 10.1155/2020/8307580 (PMC7341392; doi:10.1155/2020/8307580)
Supplement: Supplementary materials — Table 1: Clusters identified in the searchlight analysis encoding stimulated locations for acupuncture and touch stimulation. [file 8307580.f1.docx]

**Supplementary table**

**Supplementary Table 1.** Clusters identified in the searchlight analysis encoding stimulated locations for acupuncture and touch stimulation

| **Session** | **Task** | **Location** | **Alpha** | **Size of clusters** | **MNI coordinates of peak voxel** | | |
| --- | --- | --- | --- | --- | --- | --- | --- |
|  |  |  |  |  | **x** | **y** | **z** |
| **Acupuncture** | **4 points classification** | Right SI | < 0.01 | 12 | 40.2 | -36 | 55.5 |
|  |  | Right intraparietal sulcus | < 0.05 | 3 | 43.8 | -36 | 45 |
|  | **2 body parts classification** | Right SI | < 0.01 | 22 | 36.8 | -46.5 | 59 |
|  |  |  | < 0.05 | 3 | 15.8 | -43 | 62.5 |
|  |  | Right SI, MI | < 0.01 | 14 | 12 | -32.5 | 69.5 |
|  |  | Right paracentral lobule | < 0.01 | 6 | 1.8 | -25.5 | 62.5 |
|  |  |  | < 0.01 | 4 | 8.8 | -15 | 69.5 |
|  |  | Right superior frontal gyrus | < 0.05 | 3 | 1.8 | -18.5 | 55.5 |
| **Touch** | **2 body parts classification** | Right SI | < 0.01 | 38 | 15.8 | -39.5 | 69.5 |
|  |  | Right SI, MI | < 0.01 | 27 | 33.2 | -32.5 | 55.5 |
|  |  | Right MI | < 0.05 | 3 | 22.8 | -29 | 66 |
|  |  | Right paracentral lobule/superior frontal gyrus | < 0.01 | 5 | 8.8 | -18.5 | 69.5 |
|  |  | Right paracentral lobule | < 0.01 | 4 | 1.8 | -46.5 | 62.5 |
|  |  |  | < 0.05 | 3 | 26.2 | -32.5 | 62.5 |

Clusters of brain regions which were identified as most contributing voxels to the searchlight analysis. The results were identified using an uncorrected voxel-wise threshold of p < 0.001, and corrected for FWE at a significance threshold of alpha < 0.05. The cluster extent threshold was determined with 10000 iterations of a Monte Carlo simulation.

MI: primary motor cortex; MNI: Montreal Neurological Institute; SI: primary somatosensory cortex
